# Peripheral Regional Anesthesia for Awake Emergency Upper Limb Trauma Surgery in an Adult Patient With Fontan Physiology

**DOI:** 10.1155/cria/9525591

**Published:** 2025-04-09

**Authors:** Patrick N. Wiseman, Molly Featherstone, Noreen Dowd, Aislinn Sherwin

**Affiliations:** Department of Anaesthesiology, St. James's Hospital, Dublin, Ireland

**Keywords:** fontan circulation, peripheral nerve block, regional anaesthesia, supraclavicular block, trauma surgery

## Abstract

**Background:** The Fontan procedure is the principal technique used in the surgical palliation of a range of congenital heart defects involving a single functional ventricle. With improvements in surgical technique and medical management, patients with Fontan physiology are surviving longer and a growing number are now presenting for noncardiac surgery in adulthood. The Fontan physiology provides a unique challenge for anaesthesiologists managing their perioperative care. This case report explores this challenge further and emphasises the benefits which regional anaesthesia can provide in the management of these complex patients.

**Case Presentation:** We report the successful use of supraclavicular brachial plexus and intercostobrachial nerve blockade for awake surgery in a 27-year-old patient who had previously undergone a Fontan procedure at 5 years of age and now presented for emergency fixation of complex fractures of his radius, ulna and olecranon following a fall from his electric bicycle.

**Conclusions:** This case report and ensuing discussion highlight the benefits which regional anaesthesia can provide for adult patients with complex cardiac physiology, including Fontan circulation, undergoing noncardiac surgery when compared to general anaesthesia.

## 1. Introduction

The Fontan procedure is the most widely used surgical palliation for congenital heart defects with a single functional ventricle or when a biventricular repair is not possible, including left or right hypoplastic heart syndromes. Usually performed in three stages, the procedure constructs a pathway to deliver the entire vena caval blood flow directly to the pulmonary arterial system, allowing oxygenated blood from the lungs to subsequently return to a single functional ventricle [1]. A hypoplastic subpulmonic ventricle is bypassed or eliminated from the pulmonary circulation, with the pulmonary arteries receiving nonpulsatile flow directly from the vena cavae rather than pulsatile flow from the right ventricle. Some patients with suboptimal pulmonary venous return (PVR) have a small fenestration formed between the Fontan conduit and the right atrium, permitting a residual small right to left shunt, limiting caval pressure and increasing preload of the systemic ventricle, albeit at the expense of mild desaturation [1].

Improvements in surgical technique, perioperative care and long-term management have greatly improved survival, with the majority of patients now surviving well into adulthood [2]. 80% of patients undergoing the Fontan procedure today can expect a 30-year survival, resulting in an increasing incidence of patients with Fontan physiology presenting for noncardiac surgery in the adult setting [3]. The perioperative management of these patients presents a unique challenge for anaesthesiologists, with each warranting an individualised approach to their care. Regional anaesthesia, when possible, has been cited as a beneficial alternative to general anaesthesia for these patients [2]. This report describes the successful anaesthetic management of emergency upper limb trauma surgery in a patient with Fontan physiology using peripheral regional anaesthesia. This article adheres to the applicable CAse REport (CARE) guidelines (completed CARE checklist available in Supporting Information (available here)).

## 2. Case Presentation

A 27-year-old male presented via the emergency department at 0210 h with complex fractures of his left radius, ulna and olecranon after falling from his electric bicycle. His elbow and forearm were grossly deformed and tensely swollen and he was deemed at high risk of developing compartment syndrome due to the presence of pain on passive finger extension. He was listed for emergency surgery, requiring open reduction and internal fixation of his fractures and tension band wiring of his olecranon.

The patient's medical history was notable for significant congenital heart disease, with neonatal diagnoses of a hypoplastic right ventricle, tricuspid atresia, pulmonary atresia and pulmonary trunk hypoplasia. He had previously undergone a three-stage correction procedure. Initially, a Blalock-Taussig shunt was formed to increase pulmonary blood flow, followed by a Glenn procedure to redirect the drainage of the superior vena cava directly into the pulmonary arterial system. At age five, he underwent an extracardiac, fenestrated Fontan procedure, completing the formation of a univentricular circulation using the left ventricle. Other than his cardiac history, the patient had no other comorbidities. His only medication was long-term warfarin therapy which he had stopped taking 3 days prior to presentation due to a lack of supply. Functionally, he was able to swim regularly and lift light weights. His room air oxygen saturations were 94% and preoperative electrocardiogram showed normal sinus rhythm. A bedside echocardiogram revealed an estimated left ventricular ejection fraction of 35%–40%. Preoperative laboratory tests revealed a Hb of 15.8 g/dL, INR of 1.2 and normal renal function.

Discussions were undertaken with the patient and his cardiologist to confirm his previous cardiac surgeries and current physiology. An anaesthetic management plan was then formulated by the general anaesthesiology, cardiac anaesthesiology, orthopaedic and cardiac surgery teams, considering all relevant risks and benefits. Regional anaesthesia with conscious sedation as required was the anaesthetic approach chosen. In the event of block failure, general anaesthesia with the cardiac anaesthesiology team was planned.

The patient's surgery was performed in the emergency theatre 10 hours after his presentation. He remained fasting for this period, but continued to sip water until attending theatre in line with the hospital's “sip-til-send” protocol. A right radial arterial line was placed and AAGBI monitoring, including end-tidal carbon dioxide monitoring, was applied throughout. Using the linear probe (frequency 6–15 MHz) of a Sonosite Edge II ultrasound machine and a 50 mm Pajunk Sonoplex II block needle, a supraclavicular brachial plexus block was performed, supplemented by intercostobrachial nerve blockade to alleviate potential tourniquet pain. 20 mLs 0.5% levobupivacaine and 10 mLs 2% lidocaine with 1:100,000 adrenaline were injected, with good spread of local anaesthetic visualised. After confirmation of satisfactory anaesthesia 20 minutes post block, sedation was provided with 0.5 mg midazolam and a target-controlled infusion of remifentanil of between 1.0 and 1.5 ng/mL. Verbal communication with the patient was maintained throughout the surgery. Supplemental oxygen was supplied via 40% venturi mask. Arterial blood gases were performed at hourly intervals, all parameters remaining within normal limits throughout.

Surgical time was 3 hours and 15 minutes. The estimated blood loss was 220 mLs and 1 L of intravenous Hartmann's solution was administered intraoperatively. No discomfort was felt by the patient and no additional analgesia or sedation was required. The surgical correction was successful, with postoperative x-rays showing satisfactory alignment of all fractures. His postoperative pain was managed with regular paracetamol and ibuprofen, commencing the evening of surgery. Rescue analgesia was prescribed in the form of oxycodone immediate-release 5 mg PO4 hourly PRN and was administered by ward nursing staff as required. The block wore off the morning after surgery, approximately 20 h after block performance, and he did not experience rebound pain. The patient did not require any breakthrough analgesia before the block wore off. He then received three 5 mg doses of oxycodone IR on postoperative day 1 and two on postoperative day 2, and no further doses thereafter. LWMH was commenced on day one postoperatively, warfarin was restarted on day four and the patient was discharged home on day six. Prior to discharge, he reported high levels of satisfaction with his anaesthetic and surgical management. He was followed up by the hospital's warfarin clinic until his INR was in the therapeutic range of 2-3 and then recommenced his preoperative warfarin regimen.

## 3. Discussion

Adults with Fontan physiology present a unique challenge in the perioperative setting, with up to 31% of those undergoing general anaesthesia for noncardiac surgery experiencing perioperative complications [4]. Fontan physiology exhibits a number of characteristics which make the safe provision of general anaesthesia more challenging, a full understanding of which is essential to ensure optimal anaesthetic management [2].

Fontan circulation allows nonpulsative blood flow from the systemic venous system to return directly to the pulmonary arteries and then to the left atrium down a pressure gradient, with no active pumping of blood through the lungs. This is made possible by a higher systemic venous pressure in the Fontan circulation than in the atrium. Ideal pressures in a young patient with Fontan circulation are central venous pressure (CVP) of 10–15 mmHg and left atrial pressure of 5–10 mmHg, giving a transpulmonary driving pressure of approximately 5–8 mmHg [2]. Thus, pulmonary blood flow depends on CVP, PVR, functional left atrial pressure and single ventricular end-diastolic pressure. Maintaining appropriate CVP, lowering PVR and single ventricular end-diastolic pressure, and optimizing systemic blood pressure, which acts as an afterload, are essential for maintaining cardiac output in the Fontan circulation [2].

Many patients exhibit a number of multisystem comorbidities which impact their anaesthetic management [2]. Respiratory considerations are important in planning perioperative airway and ventilatory management. Restrictive respiratory pathology is common among patients with Fontan physiology, arising from both extrinsic restriction, due to respiratory and skeletal muscle weakness, and intrinsic lung pathology due to pulmonary hypoplasia caused by reduced pulmonary blood flow in utero [1]. Noncardiopulmonary sequelae often exhibited by adult Fontan patients include renal dysfunction, liver dysfunction and protein-losing enteropathy. Thrombosis is common, occurring in up to 30% of patients, with hypercoagulability resulting from enhanced platelet activation, impaired fibrinolysis, relative stasis of nonpulsatile pulmonary blood flow and polycythaemia due to chronic cyanosis [5]. Many patients will be prescribed long-term anticoagulation, the discontinuation of which will have to be managed in the perioperative setting.

Given these physiological complexities, regional anaesthesia has been cited as a beneficial alternative to general anaesthesia for adult Fontan patients undergoing noncardiac surgery for a number of reasons. Most general anaesthetic agents cause a reduction in sympathetic tone, leading to a decrease in systemic vascular resistance (SVR), venous return and cardiac output. Intravenous anaesthetic agents, including propofol and midazolam, decrease SVR and depress myocardial contractility. Ketamine can also prove problematic for Fontan patients as it increases PVR and myocardial oxygen demand. High concentrations of volatile agents also decrease SVR and myocardial contractility and should be avoided, with volatiles also carrying the risk of inducing cardiac arrythmias in susceptible patients [6]. The vasodilation which occurs with most general anaesthetic agents also renders patients more susceptible to hypothermia, which increases PVR and can in turn reduce venous return and cardiac output [7]. Arguably, many of these factors are less likely to be encountered during regional anaesthesia, particularly peripheral nerve blockade, as it is less likely to negatively impact the SVR, PVR and myocardial contractility and more likely to maintain venous return and cardiac output [8].

A further advantage of regional anaesthesia is the maintenance of spontaneous ventilation, provided hypercarbia and hypoxia are avoided. Regional anaesthesia allows spontaneous breathing, resulting in a low intrathoracic pressure and low PEEP, promoting pulmonary blood flow and venous return [9].

In contrast, the increase in PVR which occurs with positive pressure ventilation during general anaesthesia can reduce pulmonary blood flow, venous return and cardiac output [10].

It is important that fasting times are minimised and dehydration is avoided in the perioperative period. Gradient-driven pulmonary flow, and thus cardiac output, are dependent on volume status and preload. We limited preoperative fasting for our patient using the hospital's ‘sip till send' protocol. Intravenous fluids should be administered where necessary, while an early postoperative return to oral intake and minimising medications which cause nausea and vomiting allows patients to regulate their own fluid intake [1]. Regional anaesthesia usually allows an earlier reintroduction of enteral intake than general anaesthesia and reduces the amount of nauseating postoperative opioids required. Regional anaesthesia can produce relative hypovolaemia however, thus careful fluid management is required regardless of anaesthetic technique chosen.

A principal advantage of regional anaesthesia is excellent analgesia in the immediate postoperative period. Preventing severe postoperative pain decreases sympathetic drive, reducing the risk of myocardial injury in these susceptible patients [7]. Effective pain management, as achieved in this case, also facilitates normal breathing patterns postoperatively, reducing the development of respiratory complications [1]. Hypoxia, hypercarbia and acidosis will increase PVR and cause a resultant drop in venous return and cardiac output, so avoiding these complications is paramount. Regional anaesthesia also reduces the amount of opioid analgesia required, which can avoid problematic postoperative respiratory depression [1].

An increasing number of adults with Fontan physiology will continue to present for noncardiac surgery in the coming years. In the anaesthetic management of adult Fontan patients, careful preoperative assessment, a full understanding of the individual patient's physiology, appropriate intraoperative monitoring and an anaesthetic plan tailored to the individual are critical. The numerous benefits of using peripheral regional anaesthesia should be carefully considered and prioritised in this group of patients where possible.

## Data Availability

Data sharing is not applicable to this article as no new data was created or analysed during this study.

## Supporting Information

Please find attached the following Supporting Information.

Completed CARE Checklist.

Patient Consent Form (blank copy, completed copy available on request).
