# Supplementary material for: Peripheral Regional Anesthesia for Awake Emergency Upper Limb Trauma Surgery in an Adult Patient With Fontan Physiology
Source: Case Rep Anesthesiol. 2025 Apr 9;2025:9525591. doi: 10.1155/cria/9525591 (PMC12003033; doi:10.1155/cria/9525591)
Supplement: Supporting Information — Additional supporting information can be found online in the Supporting Information section. [file 9525591.f1.docx]

**Patient Consent Form**

To record a patient’s consent to publication of information about them or their relative in

British Journal of Anaesthesia (BJA)

**NAME OF PATIENT:** ______________________________________________________________

**TITLE OF ARTICLE:** _________________________________________________________

_________________________________________________________________________

**CORRESPONDING AUTHOR:** __________________________________________________

**CORRESPONDING AUTHOR’S ADDRESS:** ________________________________________

_________________________________________________________________________

**MANUSCRIPT NUMBER, IF KNOWN:** _____________________________________________

I, ................................................................................................... [NAME OF PATIENT / PARENT /

GUARDIAN / RELATIVE*], give my permission to:

...................................................................................... [NAME OF HEALTH PROFESSIONAL]

to use information (including photographs) about:

................................................................................... [NAME AND RELATIONSHIP*] in the BJA published by Elsevier, such permission to extend to publication of the information by Elsevier and its licensees in all media and languages throughout the world.

*In cases where the patient has died or is incapable of giving consent, consent may be given by the next of kin. If the patient is under the age of 16, consent should be given by a parent or guardian.

**I have seen and read the material to be submitted to the journal. I understand that:**

(1) My name will not be published and **BJA** will endeavour to ensure I remain anonymous, other than in relation to identifiable photographs for which I have given consent. However,

I also understand that it is possible somebody may recognise me from the article.

(2) I understand that **BJA** is an Open Access publication, and content is made available under the terms of the Creative Commons, primarily the Creative Commons Attribution License, which permits use, distribution and reproduction in any medium, provided that the content is properly cited. This means that my information can be read, used, and built upon

by anyone around the world for free.

(3) I can change my decision to give consent to publish information about me at any time before final approval for publication by Elsevier, but once the article has been approved for publication in its final form it will not be possible to change my decision to give consent.
